# Nature of the Active Sites on Ni/CeO_2_ Catalysts for Methane Conversions

**DOI:** 10.1021/acscatal.1c02154

**Published:** 2021-08-11

**Authors:** Pablo G. Lustemberg, Zhongtian Mao, Agustín Salcedo, Beatriz Irigoyen, M. Verónica Ganduglia-Pirovano, Charles T. Campbell

**Affiliations:** † Instituto de Catálisis y Petroleoquímica (ICP-CSIC), 28049 Madrid, Spain; ‡ Instituto de Física Rosario (IFIR-CONICET) and Universidad Nacional de Rosario (UNR), S2000EKF Rosario, Santa Fe, Argentina; § Department of Chemistry, University of Washington, Seattle, Washington 98195-1700, United States; ∥ Departamento de Ingeniería Química, Facultad de Ingeniería, Universidad de Buenos Aires (UBA), Ciudad Universitaria, C1428EGA Buenos Aires, Argentina; ⊥ Instituto de Tecnologías del Hidrógeno y Energías Sostenibles (ITHES, CONICET-UBA), Ciudad Universitaria, C1428EGA Buenos Aires, Argentina

**Keywords:** Ni nanoparticles, ceria support, dry reforming, methane to methanol, selective oxidation, metal−support interaction, metal/oxide interface, particle size effect

## Abstract

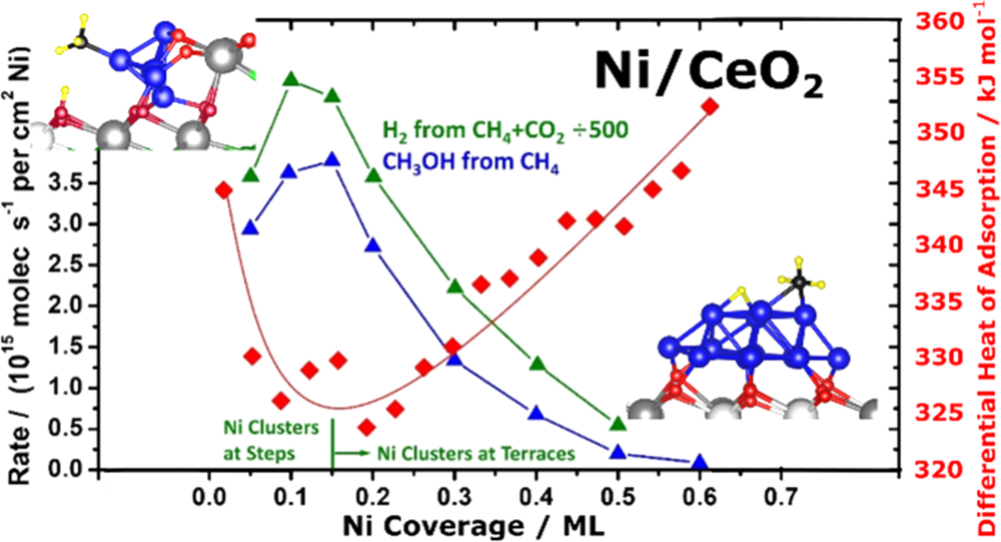

Effective catalysts for the direct conversion of methane to methanol and for methane’s dry reforming to syngas are Holy Grails of catalysis research toward clean energy technologies. It has recently been discovered that Ni at low loadings on CeO_2_(111) is very active for both of these reactions. Revealing the nature of the active sites in such systems is paramount to a rational design of improved catalysts. Here, we correlate experimental measurements on the CeO_2_(111) surface to show that the most active sites are cationic Ni atoms in clusters at step edges, with a small size wherein they have the highest Ni chemical potential. We clarify the reasons for this observation using density functional theory calculations. Focusing on the activation barrier for C–H bond cleavage during the dissociative adsorption of CH_4_ as an example, we show that the size and morphology of the supported Ni nanoparticles together with strong Ni-support bonding and charge transfer at the step edge are key to the high catalytic activity. We anticipate that this knowledge will inspire the development of more efficient catalysts for these reactions.

## Introduction

The recent dramatic increase in methane availability worldwide has inspired a surge of interest in new catalytic processes for methane conversions that could lead to major environmental and economic benefits. Dry reforming of methane (DRM) is an attractive route that could potentially utilize vast quantities of CO_2_ for its catalytic conversion to valuable syngas while simultaneously mitigating both greenhouse gases.^1−4^ Perhaps, even more impactful would be the direct catalytic conversion of methane to methanol.^5−9^ These two reactions are challenging owing to the high gas-phase stability of their reactants and the rapid deactivation through carbon deposition on high-loaded metal-based catalysts.^10−18^ It has been recently shown experimentally^3,4,9,19,20^ that small Ni nanoparticles on a ceria(111) model support promote the activation of both O–H and C–H bonds in H_2_O and CH_4_, respectively, at room temperature, with lower activation barriers than for extended metallic Ni surfaces, and promote the activation of CO_2_ at moderate temperatures. Most importantly, this type of catalyst is very active in the DRM in a clean and efficient way^3,4,20^ and in the direct conversion of methane to methanol using a mixture of oxygen and water, with a higher selectivity than ever reported for ceria-based catalysts.^9^ The activation of CH_4_ is the first and only step shared by both reactions, whereas, for example, their steps for C–O bond formation are quite different. In the partial oxidation of methane to methanol, the C–O bond is formed in the addition of chemisorbed CH_3_ to O or OH species,^9,13,21,22^ whereas in DRM, it is formed from chemisorbed CH and/or C species.^23−27^ The high activity of the Ni/CeO_2_ catalyst was mainly attributed to the highly cationic character of the interfacial Ni atoms, also reported to be the most active for water–gas shift.^19,27−31^ However, a detailed understanding of the structure and nature of the active site remains a challenge and is of paramount importance for the rational development of new or better catalysts. Herein, we report a combination of experimental measurements and density functional theory (DFT) calculations which elucidate the active site, thus hopefully enabling future designs of improved catalysts. We further reveal how this nanomaterial escapes the so-called “tyranny of linear scaling”, at least for this key step in these reactions investigated here, namely, methane’s dissociative adsorption.

## Results

### Measurements of Ni Atom Stability and Charge on Ni/CeO_2_(111) Correlate with Catalyst Activity

**Figure 1:**
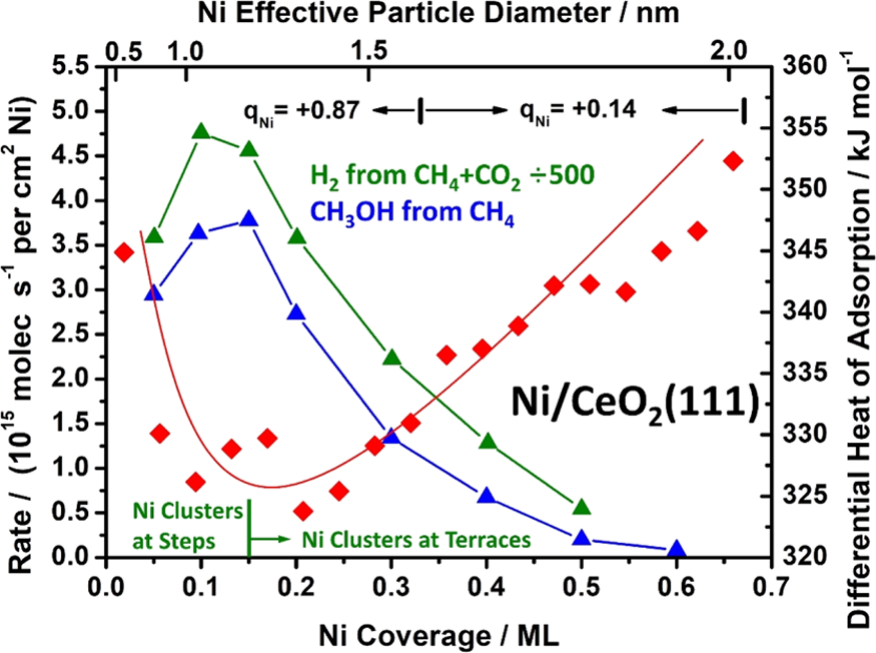
Correlation of the measured catalytic activities versus Ni coverage (and the corresponding average particle size) with Ni atom heat of adsorption for Ni on CeO_2_(111). Rates for both methane to methanol at 450 K^9^ and methane dry reforming at 650 K^3,20^ are shown. The differential heat of Ni adsorption at 300 K^32^ shows a minimum when step edges stop being populated by Ni, and Ni bonding at terraces starts to dominate, at the same coverage where both the rates maximize. This coverage corresponds to a Ni particle diameter of ∼1 nm. The average measured charge per Ni atom^32^ (shown) also drops strongly near this coverage.

Figure 1 compares the catalytic rate measurements versus Ni loading from two of the abovementioned studies^3,9,20^ (blue = CH_3_OH from CH_4_ and green = DRM) with our recent measurements of the differential heat of adsorption of Ni vapor versus coverage (red). In all cases, these are well-defined model catalysts prepared by vapor-depositing Ni onto the CeO_2_(111) support at 300 K, where the support is a nonreduced CeO_2_(111) thin film (CeO_2–*x*_, with *x* up to 0.05). These were grown by very similar recipes, on Ru(0001) for the kinetic studies and on Pt(111) for our calorimetry studies. The Ni loading is in ML (defined as the total number of Ni atoms per surface O atom, i.e., 1 ML = 7.89 × 10^14^ atoms per cm^2^). The rates are for CH_3_OH synthesis from CH_4_ (with an 8:1 mix of H_2_O + O_2_ as the oxidant) at 450 K^9^ and DRM at 650 K,^3,20^ both at very low conversions. These were originally reported as the rate per unit area of the CeO_2_ support (before Ni deposition). We have used the results of our study of this system by low-energy He^+^-ion scattering spectroscopy (He^+^ LEIS), which gave the ratio of the total Ni area to area of the CeO_2_ support versus Ni coverage,^32^ to convert these to rates per unit surface area of Ni. This is proportional to the true turnover frequency (TOF, or rate per surface Ni atom), assuming a constant number of Ni surface atoms per unit area of Ni (e.g., 1.6 × 10^15^/cm^2^ for Ni(111) type packing). We also show on the top axis of Figure 1 the average Ni particle’s diameter determined from those same LEIS data^32^ assuming flat discs with a fixed height/diameter ratio of 0.25, as suggested by scanning tunneling microscopy (STM).^35^ These STM measurements were for larger particle sizes than at the rate maximum here (∼1 nm) and possibly missed seeing many or most of the particles smaller than this size due to particle mobility and the limitations of STM imaging on oxide surfaces at the temperature used. Note that dividing the total Ni coverage (in ML) by the fractional area covered by the Ni particles measured by LEIS^32^ gives the average Ni particle thickness (in ML, or atoms per unit area, which we converted to nanometers by dividing by the number of Ni atoms per unit volume in bulk Ni(solid) and then converted to particle diameter by dividing by this height/diameter ratio (0.25)). Above 1 nm diameter in Figure 1, the Ni dispersion is approximately equal to 1 nm divided by the diameter.

As seen, the heat of adsorption initially decreases to a minimum and thereafter increases, eventually saturating at the heat of sublimation of bulk Ni(solid), 430 kJ/mol, at higher coverages than shown here.^32^ The high initial heat was attributed to the binding of Ni monomers to more stable step edges, and the initial decrease in heat was attributed to the saturation of these step edge sites so that more and more of the less favorable terrace sites are populated with increasing coverage. The heat reaches a minimum and thereafter increases due to the growth of Ni cluster size (reaching ∼2.0 nm on average at the highest coverage shown here).^32^ This increase is due to the fact that more Ni–Ni bonds are made to the new Ni atom when it adds to a larger cluster.

Most importantly, in Figure 1, the TOF for both reactions is high and nearly constant with increasing coverage until the minimum heat of adsorption is reached, after which the TOF drops rapidly. This is an outstanding example of a strong correlation between the thermodynamic stability of the metal atoms in a catalyst and its catalytic activity, in this case for two very important reactions. It clearly shows that the active sites are small clusters of Ni at step edges. There is a small increase in TOF at low coverage until it reaches a maximum just at the point where the heat of adsorption reaches a minimum (i.e., where the chemical potential of the Ni atoms in the catalyst reaches a maximum),^32^ that is, the most active sites are Ni atoms in clusters at step edges with a small size (∼1.0 nm in diameter and 0.25 nm thick^32^), wherein they have the highest Ni chemical potential.

Note that both these reactions’ rates show essentially the same dramatic and complex variation with Ni particle size (coverage). Since the dissociative adsorption of methane is the common step in both reactions, this would suggest that it is the rate-determining step (RDS) in both these reactions under the conditions measured. Although C–O bond forming is also common in both reactions, as mentioned above, in the methanol production reaction, the crucial C–O bond-forming step is the coupling between adsorbed CH_3_ with O or OH,^9,13,21,22^ whereas this step has not been considered in DFT-based mechanisms for DRM on Ni-based catalysts.^23−27^ On Ni(211), a DFT-based microkinetic model of DRM showed that O–CO bond cleavage in CO_2_ is the most rate-controlling step (i.e., the one with the highest degree of rate control) under the reaction conditions that led to the highest DRM rates.^33^ This step does not occur in the methanol production reaction. It is possible that some later C–O bond-forming step that removes adsorbed carbon atoms (or CH or CH_2_) is crucial in both reactions. No one knows yet what is the RDS on the types of sites that are shown above to be the most active for both of these reactions.

Experimentally, it has been observed that this same low coverage of Ni on CeO_2_(111) is reactive in dissociating not only CH_4_ but also H_2_O and CO_2_, that is, all the reactants involved in both reactions.^3,4,19,20^ Thus, an alternate explanation for this similarity in rates versus Ni coverage is that the active site (small Ni clusters at steps) is so much faster than larger Ni clusters in activating all reactants and for both reactions (irrespective of their rate-determining steps) that both rates just track the number of these special sites. Thus, higher Ni coverages just remove these active sites (small Ni clusters) by making them into larger clusters, so that the rates of both reactions go down with Ni coverage in almost exactly the same way. The larger (2 nm) Ni clusters must be >10-fold less active per unit area than the small (1 nm) clusters, if this explanation in true.

We also quantified the charge transfer from Ni to CeO_2_(111) versus Ni coverage using X-ray photoelectron spectroscopy (XPS).^32^Figure 1 also shows the measured average charge per added Ni atom (*q*_Ni_) in the coverage ranges shown. Upon dosing 1/3 ML, each Ni atom donates nearly one electron to the CeO_2_ (making a Ce^3+^), but above 1/3, there is very little charge transfer and the added Ni is nearly neutral.^32^ Our DFT calculations below are consistent with this, showing that small Ni clusters at steps have very cationic Ni, as well as other very special electronic structural properties, and a special ability to activate difficult catalytic reaction steps, using methane activation as an example. They also show that the surface atoms of the larger Ni clusters are nearly neutral in charge, which could explain the lower activity (see also below).

At the Ni coverage where the TOFs in Figure 1 are near their maximum (0.1–0.15 ML), our XPS studies^32^ show that a combination of the initial slight extent of reduction of the ceria and the Ni-induced reduction, leads to a surface that is 5–10% Ce^3+^. This is similar to the fraction of Ce^3+^ measured using *in situ* XPS under DRM reaction conditions at temperatures (600 and 700 K) closest to that used for measuring the TOFs in Figure 1 (650 K).^3,20^

### DFT Studies of the Stability and Electronic Character of the Ni/CeO_2_(111) Model Catalysts and the Effect of the Support Structure

**Figure 2:**
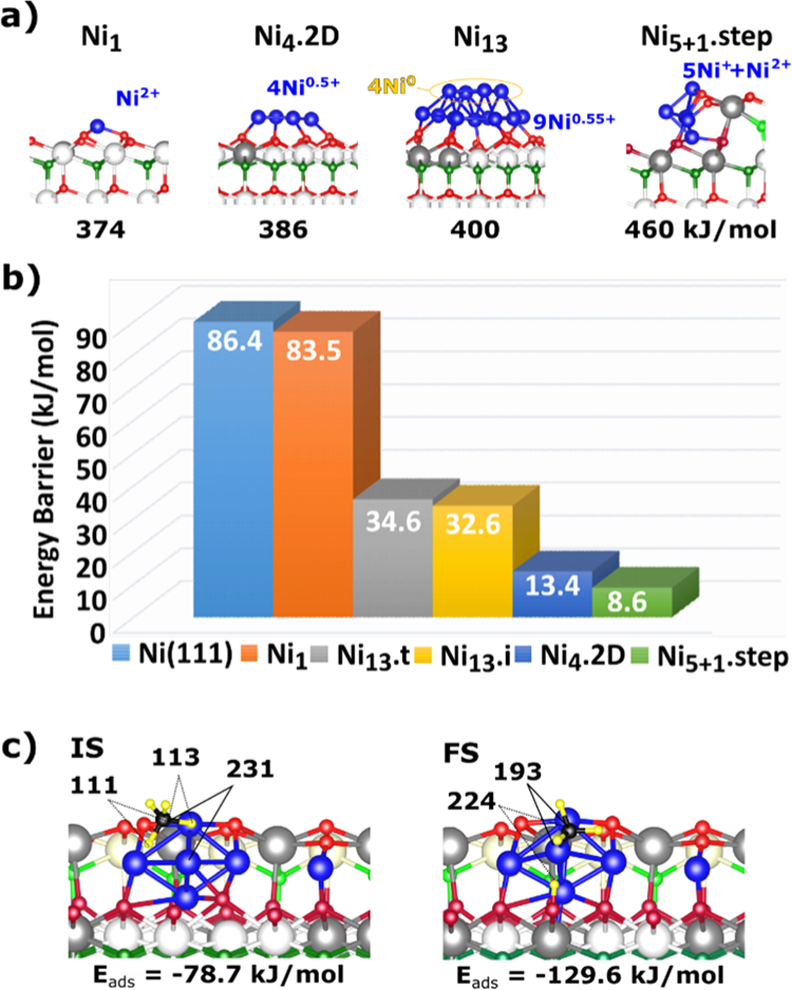
(a) Models of Ni_*n*_ (*n* = 1, 4, and 13) on CeO_2_(111) terraces and Ni_5_ + Ni_1_ (Ni_5+1_.step) at a ⟨110⟩-type step. Surface/subsurface oxygen atoms in the outermost O–Ce–O trilayer are depicted in red/green, Ce^4+^ is depicted in white, and Ce^3+^ is depicted in gray. Values of the calculated integral heat of adsorption of Ni_*n*_ species are listed below each structure in kJ/mol per Ni atom (relative to Ni gas). (Our previous results showed that DFT overpredicts these heats by ∼88 kJ/mol.^32^) (b) Activation energy barriers (non-ZPE-corrected) for the CH_4_ → CH_3_ + H reaction on these Ni_*n*_–CeO_2_ systems and for the extended Ni(111) surface. Ni_13_.t and Ni_13_.i denote dissociation on a *terrace* and at an *interface* site of the Ni_13_–CeO_2_ system, respectively (cf. Figure 1a). (c) View of the initial state (IS) and final state (FS) for the CH_4_ → CH_3_ + H reaction on the Ni_5+1_–CeO_2_ model catalysts. Selected interatomic distances (in pm) and the adsorption energy of molecularly and dissociatively adsorbed methane (in kJ/mol) are indicated.

Our DFT calculations have recently shown that Ni monomers at stoichiometric ⟨110⟩ step edges (Ni_1_.step), with a calculated heat of adsorption of 469 kJ/mol, are more strongly bound by 95 kJ/mol^32^ than on the flat CeO_2_(111) terraces (374 kJ/mol, Figure 2a). In both sites, Ni monomers bind as Ni_1_^2+^. These calculations thus predicted that decoration of the stoichiometric step with Ni species will occur before adsorption on the terraces, as found by our experiments.^32^ The maximum possible coverage of monodispersed Ni_1_ species at this ⟨110⟩ step edge is three atoms for the unit cell size used there [(5 × 3), corresponding to one Ni for every one step-edge O atoms], with two as Ni_1_^2+^ and one as Ni_1_^+^ (Figure S1). We calculated the average heat of adsorption of these three coadsorbed Ni_1_.step species (460 kJ/mol per atom) and found it to be almost the same as a single step-bound Ni_1_, still 86 kJ/mol per Ni atom more strongly bound to the step edge than isolated Ni_1_ species on the flat terrace. In our previous work,^32^ we have shown that the heat of adsorption of Ni decreases when the step becomes more and more reduced; therefore, oxygen vacancies in the steps were not considered. The size of the unit cell has been chosen to make possible the computationally demanding calculation of the minimum energy path for the dissociative adsorption of methane.

To understand how the ceria step edge affects the electronic structure, charge state, and chemical reactivity of the Ni clusters and their surface atoms, we studied several different representative Ni/CeO_2_ structures with DFT. Due to the high computational demands of these studies, we were forced to use smaller clusters than the 1 nm size shown experimentally to be the most active. We chose cluster structures which nevertheless illustrate accurately many of the essential atomic-scale features that control Ni atom stability, electronic structure, and surface reactivity, as described below. These included a small pyramidal Ni_5_ cluster (with a rhombohedral base) at the ⟨110⟩ step edge, an isolated Ni_1_ adatom, and a flat rhombohedral Ni_4_.2D cluster on the ceria terrace, for which all Ni atoms are interfacial, as well as a Ni_13_ cluster which has a two-layered 9–4 stacking structure. A structural Ni_5_ isomer was found to be less stable than the pyramidal Ni_5_ cluster by 32 kJ/mol, and consequently, it was not considered further (Figure S1). The Ni_13_ cluster has been selected as a representative model that features a compact structure that maximizes the atomic coordination, which makes it particularly likely to be energetically stable. Such a Ni_13_ cluster supported on TiC(001) has recently been used to study the effect of Ni–carbide interactions on the activation of methane.^34^

We first consider the formation of a somewhat larger Ni nanoparticle than we previously studied at the ⟨110⟩ step edge, namely, a pyramid with a rhombohedral base (Ni_5_, Figures 2a,c and S1), by adding three more Ni atoms to the fully decorated ⟨110⟩ step with three Ni_1_ species. As shown (Figure 2c), two of the original Ni atoms are incorporated into the resulting Ni_5_ cluster and one remains isolated. This resulting structure has seven Ce^3+^ ions and consists of a pyramidal Ni_5_^5+^ nanoparticle and one Ni_1_ (Ni_1_^2+^), hereinafter: Ni_5+1_.step (Figure S1, Table S6). The top atom in this pyramid is the leftmost atom shown in Figure 2a, which, importantly, enables H attachment to the support during H–CH_3_ dissociation, as shown below. This step-bound structure has a calculated integral heat of Ni(gas) adsorption that is 66–68 kJ/mol per Ni atom larger than flat Ni_6_.2D (392 kJ/mol) or Ni_6_.3D (394 kJ/mol) clusters on the CeO_2_(111) terrace.^32^ This step cluster is also more stable than the rhombohedral Ni_4_.2D cluster (which makes 2 Ce^3+^ ions, Figure 2a) and the Ni_13_ aggregate (which makes 5 Ce^3+^ ions), wherein only the four and nine Ni atoms, respectively, in direct contact with the oxide support are partially oxidized (4 × Ni^0.50+^ and 9 × Ni^0.56+^_,_ respectively), whereas the four second-layer Ni atoms in Ni_13_ retain their metallic character (Ni^0^). As previously observed,^20,28,32^ inspection of the calculated electronic structure for the Ni–CeO_2_ systems shown in Figure 1a reveals that the electronic perturbations (e.g., charge transfer) induced by the support are much stronger for Ni atoms which are directly at the Ni–ceria interface, whereas there is almost no charge transfer from the Ni atoms in the second and thicker layers of 3D nanoparticles.^29,35^ In contrast, this charge transferred by the Ni atoms is much larger for Ni aggregates at steps and extends to the second Ni layer, that is, the Ni_5+1_.step structure has a Ni_5_^5+^ pyramid with substantial charge even on the top Ni atom and more on the four Ni atoms in the base (totaling +1 charge per Ni, on average) and one Ni^2+^.

### DFT Studies of Methane Activation by Ni–CeO_2_ and Linear Scaling Relationships

There is an indisputable correlation between the highest catalytic activity for both methane dry reforming and methane conversion to methanol and the existence of small clusters of nickel dispersed at ceria steps (Figure 1). As noted above, the active low-loaded Ni–CeO_2_ systems are much faster not only for both net catalytic reactions than larger Ni clusters but also in dissociating all the reactant gases (CH_4_, H_2_O, and CO_2_). Hence, the positive effects of having small Ni clusters at ceria steps should be reflected in all steps in these reactions. In the following, we test if such sites are indeed particularly active for CH_4_ dissociation and consider the activation of the first C–H bond upon CH_4_ adsorption on various Ni/CeO_2_(111) model catalyst structures, employing the spin-polarized DFT + *U* approach. Thus, we have calculated the energy profile for methane dissociation at the Ni_5+1_.step structure shown above and compared that with those on the extended Ni(111) surface,^36^ on Ni_1_,^36^ Ni_4_.2D,^36^ and Ni_13_ clusters supported on CeO_2_(111) terraces. On the Ni_13_ cluster, two Ni sites were considered: one interfacial (i.e., at the perimeter of the Ni cluster) and one on the Ni terrace.

Figure 2b shows that the activation barrier for CH_4_ dissociation is the *lowest* among all the clusters and sites modeled for the Ni atom at the apex of the Ni_5_ pyramid at a ⟨110⟩ step (8.6 kJ/mol). For the small Ni_4_.2D cluster on the CeO_2_ terrace with all the Ni atoms being interfacial, the activation barrier for the CH_4_ → CH_3_ + H reaction is larger by 4.8 kJ/mol, whereas for interfacial and terrace sites on the Ni_13_ cluster, it is larger by up to 26 kJ/mol.

**Figure 3:**
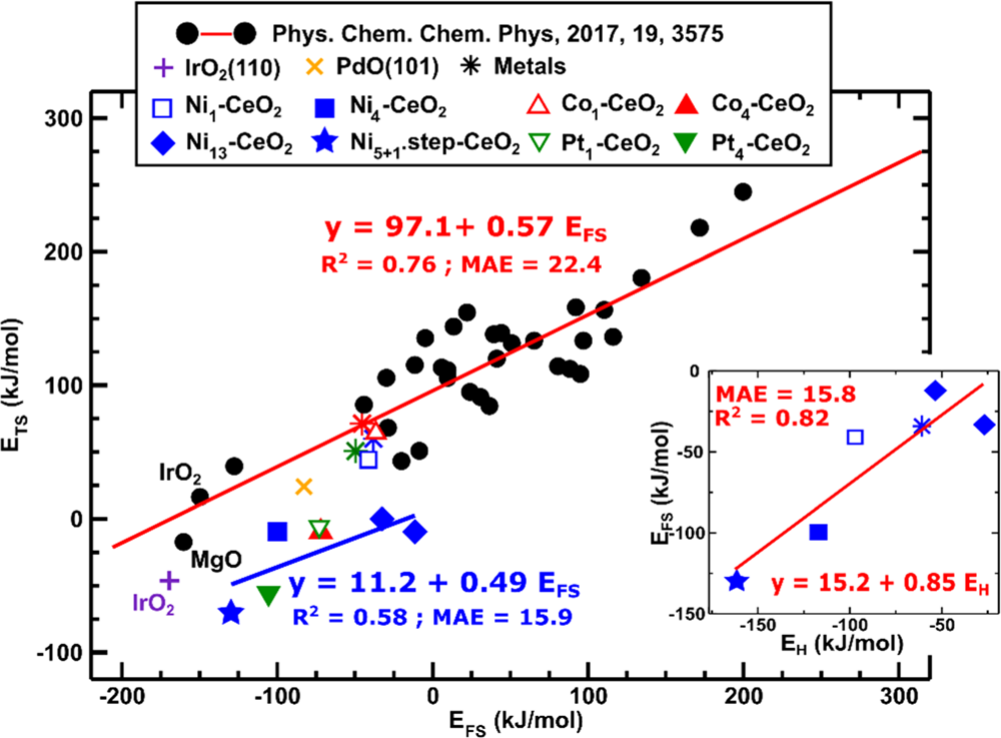
Brønsted relation between the calculated TS and FS energies for the surface-stabilized pathway in methane dissociation on a wide variety of materials. The black dots are for the many systems presented in ref (38), and the red line is the best linear fit reported for these data: *E*_TS_ = 0.57*E*_FS_ + 97.1. The purple (+) and yellow (×) symbols are for IrO_2_(110) and Pd(101) presented in refs (45), (46), and (47), respectively. The green, red, and blue symbols correspond to the *E*_TS_ and *E*_FS_ values for M_1_ atoms and M_4_ clusters (M = Pt, Co, and Ni) on the CeO_2_(111), respectively. Values for the extended Pt(111), Co(0001), and Ni(111) surfaces, as in ref (36), and for the Ni_13_ clusters on terraces and Ni_5+1_ at steps are shown. The blue line is the best linear fit for the data corresponding to the Ni_4_.2D, Pt_4_.2D, Co_4_.2D, and Ni_13_ clusters on terraces and Ni_5+1_ at steps. The inset shows the linear relation between the FS binding energy (*E*_FS_) and the hydrogen binding energy (*E*_H_) for the nickel-containing catalysts only.

To shed light on the origin of the activity of ceria-supported Ni clusters, we recall that when dealing with the activation of methane, several descriptors and scaling relations have been examined for the cleavage of the first C–H bond in the hydrocarbon.^37−43^ In general, these descriptors and scaling relations provide guidelines to compare and predict the performance of potential new catalysts with that of the existing materials used for C–H bond activation.^37,38,41^ Computational volcanos have become important tools in the design of catalysts, and scaling relations are often used in constructing such volcanos and generally considered to have good accuracy.^38,42,44^ In the case of methane activation, volcano plots have been presented for metal and/or oxide systems.^38,42,44^ For surface-stabilized methane activation pathways, Latimer et al.^38^ have proposed a linear Brønsted relation between the energy of the transition state (TS) structure for methane activation, *E*_TS_ (referenced to gas-phase CH_4_ and the clean surface), and that of the FS, *E*_FS_ = *E*_CH_3_+H_, according to which stronger CH_3_ + H binding energies correspond to lower *E*_TS_ energies, as shown in Figure 3. This model (the red line) can describe a wide range of materials such as CaO, MgO, PdO, doped MoS_2_, and rutile oxides in addition to clean and O- and OH-promoted metals (black dots in Figure 3) with reasonable accuracy. Recently, we have discussed the corresponding results for M_1_ atoms and M_4_.2D clusters (M = Pt, Co, and Ni) on the CeO_2_(111) and on the extended Pt(111), Co(0001), and Ni(111) surfaces.^17^ We now include in Figure 3 these results, as well as those for the Ni_13_ cluster on the CeO_2_(111) terrace and for the Ni_5+1_.step at the ⟨110⟩-type step.

Inspection of Figure 3 reveals that the TS energies for the extended Pt, Ni, and Co surfaces and the Ni and Co monomers on CeO_2_(111) agree well with the Brønsted relation of Latimer et al. However, the TS energies for the Ni clusters on CeO_2_(111) are all much lower than its prediction (by 28 to 102 kJ/mol, blue filled square and rhombuses in Figure 3, for values, see Table S2). Importantly, the Ni–CeO_2_ system for which the final CH_3_ + H state is most strongly bound, namely, the Ni_5+1_.step at ⟨110⟩ steps (blue filled star), with a FS adsorption energy value of −129.6 kJ/mol (Table S2), has the TS structure of the lowest energy. It is ∼50 kJ/mol below the lowest black point from the original data set of Latimer et al.^38^ and ∼57 kJ/mol below its prediction. We note that large deviations below the predicted *E*_TS_ values also exist for other ceria-supported metal clusters such as Pt_4_.2D clusters on the CeO_2_(111) terrace (Δ*E*_TS_ = 84.5 kJ/mol, green filled triangle in Figure 3).^36^

These deviations of the CeO_2_-supported Pt nanoparticles from the previous Brønsted relation have recently been explained as a combined effect of the size and morphology of the nanoparticles and strong metal–support interactions, which lead to the stabilization of both the CH_4_ molecule (−70 kJ/mol) and the CH_3_ + H dissociation product (−105.6 kJ/mol), producing active and stable catalysts for methane activation under very mild conditions.^36^ The Pt_4_.2D cluster on the CeO_2_(111) terrace provides a path for methane activation with a low activation energy barrier of 14.4 kJ/mol that does not involve cooperative interactions between Pt and an O center of the support.

This raises the questions: Can the high reactivity of Ni–ceria systems for methane activation be explained in the same way? Is the Ni_5+1_.step system unique in some way? When compared not only with all the other Ni–ceria systems investigated (cf. Table S2) but also with Pt_4_.2D–ceria,^36^ it has the lowest energy barrier for the activation of CH_4_ (8.6 kJ/mol, Figure 2b), and its final CH_3_ + H state (FS) is the most strongly bound (−129.6 kJ/mol, Figure 3). Its initial adsorbed CH_4_ state (IS) is also the most strongly bound (−78.7 kJ/mol). Elucidating the origin of the strong binding of molecularly and dissociatively chemisorbed CH_4_ at the Ni_5+1_.step may provide crucial knowledge on the nature of the active site that enables further improvements on the activity of metal-based catalysts for methane activation.

The dissociation product for the Ni_5+1_.step, as shown in Figure 2c with CH_3_ and H bound to the Ni_5_ cluster and to the ceria surface, respectively, reflects that Ni and surface lattice O atoms work in a *cooperative way* to dissociate CH_4_ molecules. Such adsorption sites with adjacent Ni and lattice O atoms exist in the low-loaded (∼0.15 ML) active Ni–CeO_2_ catalysts for methane conversion^3,4,9^ since, as discussed above (Figure 1), for such loadings, Ni binds at step-edge sites. An alternative FS in which both the CH_3_ and H species are bound to the Ni_5_ cluster is less stable by 123 kJ/mol (Figure S2), and the activation barrier to reach that dissociation product is higher by 75 kJ/mol than that for the path along which lattice O facilitates the dissociation of CH_4_ (8.6 kJ/mol). This Ni_5+1_.step–CeO_2_ system is special in this respect. The lowest-barrier path for CH_4_ dissociation for the Ni_4_.2D and Ni_13_ and Pt_4_.2D and Co_4_.2D clusters on CeO_2_(111) terraces, which also occur with relatively small barriers of 3 to 35 kJ/mol (Table S2 and ref (36)), occurs exclusively on the metal atoms and not with the cooperativity found here for this Ni_5+1_.step–CeO_2_ system whereby the H product is bound instead to a lattice O.

We note that a cooperative pathway has also been discussed for dissociation on a Ni_1_ adatom on a CeO_2_(111) terrace (Figure S2),^3,20^ but in this case, the binding of the dissociation product is weaker by 88.3 kJ/mol compared to Ni_5+1_.step (Table S2). Comparison of the CH_3_ + H binding structures for the Ni_1_–CeO_2_(111) and Ni_5+1_.step–CeO_2_ systems (Figure S3) reveals that the Ni_1_ species that adsorbs on a hollow site coordinated to three surface oxygen atoms (Figure 2a) is lifted upon adsorption of the CH_3_ species, becoming twofold coordinate instead, which destabilizes the structure. In addition, the distance between the C of the CH_3_ group on a Ni site and the H of the formed OH on the ceria support (Figure S3) is by about a factor of 2 smaller (224 pm) for the Ni_5+1_.step–CeO_2_ system as compared to Ni_1_ on the ceria terrace (443 pm).

To further stress the argument that it is the cooperativity between the Ni and the ceria support that makes the Ni_5+1_.step–CeO_2_ system special in terms of the ability to stabilize the CH_3_ + H products of the first hydrogen abstraction from CH_4_, we considered separately the binding of the CH_3_ group and that of H on all Ni–ceria systems investigated (Figures S4 and S5). We observed that CH_3_ alone on the Ni_4_.2D–CeO_2_ system is more strongly bound by 14.7 kJ/mol than on Ni_5+1_.step–CeO_2_. However, when the full FS (coadsorbed CH_3_ and H) is considered, which for the Ni_5+1_.step–CeO_2_ system implies the formation of OH, the relative stabilities are reversed, with a FS for Ni_5+1_ on ceria steps that is by 29.8 kJ/mol more stable than that for Ni_4_.2D clusters on CeO_2_(111) terraces (Table S2).

The energy of adsorbed atomic H (wrt 1/2 H_2_), calculated by eliminating the CH_3_ species from the CH_3_ + H FS of all Ni–ceria systems investigated (Figure S4), is the strongest (−162.4 kJ/mol) on an O atom at a terrace site neighboring the step of the Ni_5+1_.step–CeO_2_ system. The inset in Figure 3 shows the existence of a strong linear correlation between the energy of the FS and the binding energy of atomic H, *E*_H_. Hence, the affinity for H can be used as a probe of the local reactivity toward hydrogen abstraction from CH_4_. Here, it is important to note that FS structures where the H is on the CeO_2_ in the form of an OH species are generally more stable than those where the H species remain on the Ni cluster. However, in most clusters, all such ceria sites are too far from the Ni-bound CH_4_ to stabilize the TS for C–H cleavage. The Ni_5+1_.step–CeO_2_ has a special geometry in that respect, which favors the direct “landing” of the abstracted H on the ceria support. In its TS structure, the distance between the H and the lattice O where the O–H bond forms is 252 pm (Figure S3), whereas it is 393 pm for the Ni_4_ cluster (cooperative pathway, see Figure S3). Consequently, the activation barrier for this process is 115 kJ/mol higher than that of the path that ends with H on the Ni_4_ cluster [128.6 vs 13.4 kJ/mol, see Figure S2].

Note that the strongest CH_3_ + H binding energy for Ni_5+1_.step–CeO_2_ among all Ni–ceria systems investigated corresponds to the lowest *E*_TS_ energy. This is consistent with a linear Brønsted relation for this subset of systems in Figure 3 (the blue line), which is steeper in slope and lies well below the original Brønsted relation there. To shed light into the origin of the large deviation of Ni_5+1_.step–CeO_2_ from the *E*_TS_ values predicted by that original Brønsted relation (Δ*E*_TS_ = 56.7 kJ/mol), we inspected the interaction between CH_4_ and the Ni_5+1_.step–CeO_2_ system, that is, the IS in the CH_4_ to CH_3_ + H reaction (Figure 2c). We found that the adsorbed CH_4_ molecule is much closer to the surface as compared, for example, to CH_4_ on a Ni_1_ adatom on a CeO_2_(111) terrace (with C–Ni distances of 231 and 312 pm, respectively). For the Ni_4_.2D and Ni_13_ systems, which have activation barriers lower than about 35 kJ/mol (Figure 2b), the CH_4_ molecule also binds very close to the surface (with C–Ni distances of 212 (Ni_4_.2D), 218 (Ni_13_.i), and 228 (Ni_13_.t) pm). For Ni_5+1_.step–CeO_2_ and Ni_4_.2D–CeO_2_ and Ni_13_–CeO_2_, the direction of electron transfer is to the adsorbed CH_4_, as reflected by the increase in the Bader charge for the C atom upon CH_4_ adsorption (between 0.11 and 0.16 |e|), with respect to the gas-phase molecule (Table S4); this is not the case for CH_4_ adsorption on Ni_1_–CeO_2_. The important consequence of such a close approach is that the C–H bond that will ultimately be cleaved is already partially activated, with a substantially elongated bond distance, whereas the variation in the other three C–H bonds is almost negligible (Figure S3). This is crucial for the facile dissociation of the first C–H bond on the low-loaded Ni–CeO_2_ systems. The case of the Ni_5+1_.step is shown in Figure 2b where the elongation of one C–H bond upon CH_4_ adsorption can clearly be seen. A similarly strong CH_4_ adsorption has been recently reported on Pt_1_/TiO_2_(110),^41^ Pt_4_/CeO_2_(111),^36^ and a two-layer-thick PdO(101) film on Pd(100) as compared to a one-layer film.^48^ The elongation of one C–H bond upon CH_4_ adsorption is reported for all these systems and is accompanied by a significant reduction of the activation barrier for CH_4_ dissociation. We further note that for many of the systems in the original set in ref (22), CH_4_ is barely or not adsorbed. However, as already mentioned, this is *not* true for some of the metal/CeO_2_ systems (nor for IrO_2_(110) and Pd(101)), for which the binding of the IS is substantial with one C–H bond partially activated. The best linear fit for the *E*_TS_ versus *E*_FS_ data corresponding to the Ni_4_.2D, Pt_4_.2D, Co_4_.2D, and Ni_13_ clusters on terraces and Ni_5+1_ at steps is *E*_TS_ = 0.49*E*_FS_ + 11.2 (the blue line in Figure 3). We also calculated the best linear fit for the *E*_Barrier_ versus *E*_Reaction_ data corresponding to the Ni_4_.2D, Pt_4_.2D, Co_4_.2D, and Ni_13_ clusters on terraces and Ni_5+1_ at steps, *E*_Barrier_ = 0.28*E*_Reaction_ + 26.6. The comparison of these two linear fits (*E*_TS_ vs *E*_FS_ and *E*_Barrier_ vs *E*_Reaction_) indicates that about 60% of the slope of the *E*_TS_ versus *E*_FS_ regression line is due to a “true” Brønsted relation and about 40% is due to the fact that the FS energy tracks to some extent the IS energy. This 40% is due to the simple fact that metal sites that strongly bind one small C/H containing adsorbate also tend to bind other C/H-containing adsorbates strongly.

**Figure 4:**
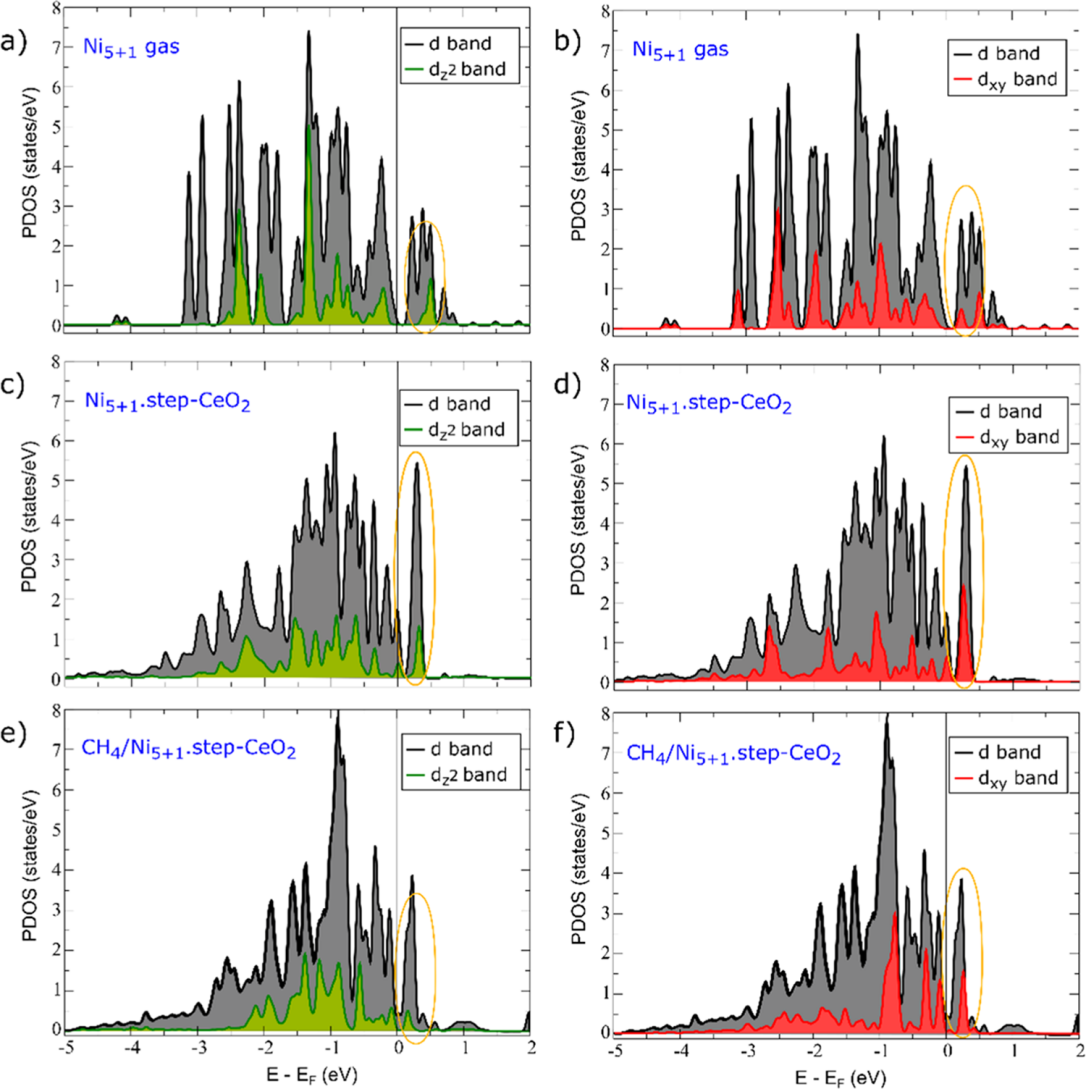
Projected density of states (PDOS) onto the d-states of the Ni atom at the apex of the Ni_5_ pyramid of the Ni_5+1_.step system. The energy zero is the Fermi level (*E*_F_). The green and red filled curves are the corresponding d*z*^2^ and d*xy* projected density of states. (a,b) Results for a free-standing Ni_5+1_ aggregate resulting from the removal of the CeO_2_ support from Ni_5+1_.step–CeO_2_, without further geometry optimization, (c,d) results for the Ni_5+1_.step–CeO_2_ system, and (e,f) those for the CH_4_ adsorption on Ni_5+1_.step–CeO_2_. The empty states close to *E*_F_ are highlighted.

To elucidate the reason why CH_4_ can get so close to the active Ni_5+1_.step–CeO_2_ system, we inspect first the consequences of the existence of strong metal–support interactions on the d-states of the Ni atom over which CH_4_ dissociates. Figure 4 shows the projected density of states (PDOS) onto the d-states of the Ni atom at the apex of the Ni_5_ pyramid for three different cases, namely, the free-standing Ni_5+1_ aggregate resulting from the removal of the CeO_2_ support from Ni_5+1_.step–CeO_2_, without further geometry optimization, and the Ni_5+1_.step–CeO_2_ and the CH_4_/Ni_5+1_.step–CeO_2_ systems. The detailed analysis of the PDOS (Table S5) reveals that two states, namely, d*z*^2^ and d*xy*, become less occupied upon adsorption of the Ni_5+1_ aggregate onto the ⟨110⟩ ceria step. The consequence of such a ligand effect is that the Pauli repulsion to the methane’s frontier orbital is reduced and the molecule is able to move closer to the surface. These states are then occupied upon CH_4_ adsorption as measured by the decrease in the number of empty d*z*^2^ and d*xy* states in the CH_4_/Ni_5+1_.step–CeO_2_ system (Figure 4, Table S5). The electronic perturbation (especially this electron transfer) induced by the binding of Ni to oxygen atoms of the ceria support is important for reactivity toward the first hydrogen abstraction from CH_4_ in the Ni_5+1_.step–CeO_2_ system.

We note that a CH_4_ molecule that approaches a Ni_1_^2+^ adatom on a CeO_2_(111) terrace finds the d*z*^2^ state occupied (Figure S8, Table S5), and thus, the repulsion to the frontier methane orbital is larger as compared to the Ni_5+1_.step–CeO_2_ system; consequently, the CH_4_ binding is weaker, the C–H bond that will ultimately be cleaved is less elongated, and the deviation between the calculated activation energy barrier and that predicted by the original Brønsted relation is less pronounced.

## Conclusions

Single-crystal adsorption calorimetry and surface analysis measurements (LEIS, XPS, and LEED) combined with DFT calculations have allowed the nature of the active sites in Ni/CeO_2_ catalysts for important methane conversions to be identified. The heat of Ni adsorption onto CeO_2_(111) at 300 K starts from 345 kJ/mol, decreases within the first 0.15 ML to 323 kJ/mol, and increases afterward. This behavior has been correspondingly attributed to the binding of Ni monomers and small clusters to more stable step edges and the saturation of these step edge sites so that less favorable terrace sites are populated with increasing coverage (and Ni cluster size). A very strong correlation of the heat of adsorption with the catalytic rate measurements versus Ni loading for both the direct conversion of CH_4_ to CH_3_OH and the CH_4_ dry reforming with CO_2_ over the Ni–CeO_2_(111) catalyst reveals that the activity for both reactions is high and nearly constant with increasing coverage until the minimum heat of adsorption is reached, after which the TOF drops rapidly. This clearly shows that the active sites are small, highly cationic clusters of Ni at CeO_2_ step edges, with the highest Ni chemical potential. Moreover, the same coverage of Ni on CeO_2_(111) which we show here produces these active sites and was also experimentally shown to be reactive toward dissociative adsorption of all reactants in both reactions. This conclusion is supported by DFT calculations on small Ni clusters on CeO_2_(111) terraces and at the ⟨110⟩ step edge that show the lowest energy barrier for the example of CH_4_ activation for 2D Ni clusters at this CeO_2_ step edge. The calculated H–CH_3_ dissociation barrier at this active site drops ∼60 kJ/mol below a previously reported linear scaling relation. Ni–CeO_2_ interactions at the step edge lead to stabilization of both the adsorbed CH_4_ molecule and its CH_3_ + H dissociation product, producing active catalysts. By comparing with other metal–CeO_2_ systems, we show that by choosing the “right” metal–oxide combination and manipulating metal–oxide interactions, as well as controlling the structure of the ceria support and the effects of metal loading, an improved activity for methane dissociation can be obtained. Such stabilization by small, non-noble metal clusters at steps of reducible oxide surfaces suggests a promising approach to design efficient catalysts for methane conversion. Moreover, we show that cationic Ni atoms in clusters, with a small size, circumvent the existing linear energy scaling relationship for the cleavage of the first C–H bond in CH_4_, which corresponds to the discovery of catalysts following another scaling relationship. This study paves the way for a new way of thinking for the rational design of improved and stable catalysts for methane conversions, a major goal in heterogeneous catalysis.

## Methods

### Computational Methods

All electronic structure calculations were carried out using the spin-polarized DFT approach as implemented in the Vienna Ab initio Simulation Package (VASP) [vasp site, http://www.vasp.at; version vasp.5.3.5 and vasp5.4.1].^49,50^ Ce (4f, 5s, 5p, 5d, and 6s), O (2s and 2p), and Ni (3p, 3d, and 4s) electrons were explicitly treated as valence states within the projector augmented wave method^51^ with a plane-wave cutoff energy of 415 eV, whereas the remaining electrons were considered as part of the atomic core. Total energies and forces were calculated with precisions of 10^–6^ eV and 10^–2^ eV/Å for electronic and force convergence, respectively, within the DFT + *U* approach by Dudarev et al.^52^ (*U*_eff_ = *U* – *J* = 4.5 eV for the Ce 4f electrons) with the generalized gradient approximation (GGA) proposed by Perdew, Burke, and Ernzerhof.^53^ We note that questions regarding the best value for the *U* parameter are still under debate.^54−56^ Nonetheless, most DFT + *U* studies of reduced ceria-based systems agree that *U* values in the range of 4.5–6.0 eV with GGA are suitable for the description of the localization of charge driving the Ce^4+^ → Ce^3+^ reduction. However, one should bear in mind that there is, in general, no unique *U* that gives a reasonable account of all systems’ properties.^57−59^ Long-range dispersion corrections were also considered, employing the so-called DFT-D3 approach.^60,61^ For details on the models and additional computational details, see Supporting Information.

## Supporting Information Available

Models and computational details; initial, transition, and final states for the CH_4_ → CH_3_ + H reaction on ceria-supported Ni clusters; binding of CH_3_ and H; Bader charges; projected DOS; and Brønsted relation between the calculated activation barrier and reaction energy (PDF)

## Notes

The DFT data that support the findings of this study are available in Materials Cloud {https://www.materialscloud.org/home} with the identifier DOI: 10.24435/materialscloud:ks-qb. The repository contains the calculations described in Figures 2–4 and the related content in Supporting Information. The data are also available from the authors upon reasonable request.
