# Supplementary material for: Nature of the Active Sites on Ni/CeO2 Catalysts for Methane Conversions
Source: ACS Catal. 2021 Aug 11;11(16):10604–13. doi: 10.1021/acscatal.1c02154 (PMC8411779; doi:10.1021/acscatal.1c02154)
Supplement: Supplementary file 1 — cs1c02154_si_001.pdf [file cs1c02154_si_001.pdf]

Supporting Information

Nature of the Active Sites on Ni/CeO_2 catalysts for Methane Conversions

Pablo G. Lustemberg,[1,2⊥] Zhongtian Mao[3⊥], Agustín Salcedo,[4,5] Beatriz Irigoyen,[4,5] M. Verónica Ganduglia-Pirovano[1]* and Charles T. Campbell[3]*

[1]Instituto de Catálisis y Petroleoquímica (ICP-CSIC), 28049 Madrid, Spain

[2]Instituto de Física Rosario (IFIR-CONICET) and Universidad Nacional de Rosario (UNR), S2000EKF Rosario, Santa Fe, Argentina

[3]Department of Chemistry, University of Washington, Seattle, Washington 98195-1700, USA

[4]Departamento de Ingeniería Química, Facultad de Ingeniería, Universidad de Buenos Aires (UBA), Ciudad Universitaria, C1428EGA Buenos Aires, Argentina

[5]Instituto de Tecnologías del Hidrógeno y Energías Sostenibles (ITHES, CONICET-UBA), Ciudad Universitaria, C1428EGA Buenos Aires, Argentina

* Corresponding authors: Charles T. Campbell: charliec@uw.edu
M. Verónica Ganduglia-Pirovano: vgp@icp.csic.es

⊥ These authors have contributed equally to this work.

Models and Computational Details

The extended metallic Ni(111), Co(0001) and Pt(111) surfaces were modeled as previously reported, the two former ones by Liu et al.[1] and the latter by Lustemberg et al.[2]. The CeO_2(111) oxide support was modeled with the calculated ceria bulk equilibrium lattice constant of 5.485 Å. Table S1 indicates the size of the unit cell used for each Ni_n-CeO_2 model catalyst and the number of CeO_2 (O–Ce–O) tri-layers of the ceria slab. All Ni_n-CeO_2 models used in this work are shown in Figure S1 where the most representative distances are indicated in pm. In all surface models, consecutive slabs were separated by at least a 12 Å-thick vacuum layer to avoid interaction between the slabs and their periodic images.

Monkhorst-Pack grids[3] have been used for *k*-point sampling as listed in Table S1. All atoms in the two (one) bottom layers of Ni_1-CeO_2 (Ni_4, Ni_{13} and Ni_{5+1}.step) were kept fixed at their optimized bulk-truncated positions during geometry optimization, whereas the rest of the atoms were allowed to fully relax. The integral heat of adsorption of Ni gas atoms forming Ni_n clusters on the CeO_2(111) support was calculated at 0 K as $E_{ads} = -1/n\ [E(Ni_n\text{-}CeO_2) - E(CeO_2) - n*E(Ni_{atom})]$ where $E(Ni_n\text{-}CeO_2)$ and $E(CeO_2)$ are the total energies of the Ni_n-CeO_2(111) and CeO_2(111) surfaces, and $E(Ni_{atom})$ is that

of a gas-phase Ni^0 atom in the d^9s^1 configuration, calculated with a (12×11×16) $Å^3$ periodic cell and the Γ-point. The lattice parameter of bulk fcc Ni was optimized (Ni_{bulk}: 3.48 Å, DFT+D3), using a Monkhorst-Pack grid with (15×15×15) k-point sampling of the Brillouin zone, and the heat (enthalpy) of sublimation of bulk Ni (bulk cohesive energy) was calculated to be $\Delta H_{sub,Ni}^{calc}$ = 518 kJ/mol. These are in good agreement with prior results.[4,5]

The adsorption energy of methane was calculated according to the following equation for the example of the dissociative adsorption on the $Ni_n/CeO_2(111)$ system: $E_{ads} = E[(CH_3+H)/Ni_n\text{-}CeO_2(111)] - E[Ni_n\text{-}CeO_2(111)] - E[CH_{4gas}]$, where $E[(CH_3+H)/Ni_n\text{-}CeO_2(111)]$ is the total energy of the methyl and hydrogen species co-adsorbed on the surface, $E[Ni_n\text{-}CeO_2(111)]$ is the total energy of the surface without the adsorbate, $E[CH_{4gas}]$ is the energy of the methane molecule in gas phase.

To locate transition state (TS) structures, we employed the climbing image nudged elastic band method (CI-NEB)[6] with nine images for each reaction pathway. For all the TS reported in this work, we have found only one imaginary frequency, and the full geometry optimizations starting from its back and forward nearest configurations (along the reaction path) ended in a non-dissociated and dissociated state, respectively.

In the calculated potential energy profiles, the energy barrier, $E_{Barrier} = E_{TS} - E_{IS}$, equals the difference between the energy of the transition state, E_{TS}, and the initial (molecularly chemisorbed) state, E_{IS}, whereas the effective or apparent energy barrier is given by the energy of the transition state, E_{TS}, referenced to gas-phase CH_4 and the clean surface.

<table>
<tr><th>Catalyst</th><th>Unit cell periodicity</th><th>k-pts mesh</th><th>Number of ceria trilayers</th></tr>
<tr><td>Ni(111)</td><td>3 × 3</td><td>5 × 5 × 1</td><td>5</td></tr>
<tr><td>Ni_1-CeO_2</td><td>2 × 2</td><td>3 × 3 × 1</td><td>4</td></tr>
<tr><td>Ni_4-CeO_2</td><td rowspan="2">3 × 3</td><td rowspan="2">2 × 2 × 1</td><td rowspan="9">2</td></tr>
<tr><td>Ni_{13}-CeO_2</td></tr>
<tr><td>Ni_{5+1}.step-CeO_2</td><td rowspan="7">5 × 3</td><td rowspan="7">1 × 2 × 1</td></tr>
<tr><td>Ni_{4+1}.step-CeO_2</td></tr>
<tr><td>Ni_3.step-CeO_2</td></tr>
<tr><td>Ni_3.trimer.step-CeO_2</td></tr>
<tr><td>Ni_{3+1}.step.CeO_2</td></tr>
<tr><td>Ni_4.step.CeO_2</td></tr>
<tr><td>Ni_5.step.CeO_2</td></tr>
</table>

Table S1. Computational setup employed for each Ni_n-CeO_2 model catalyst.

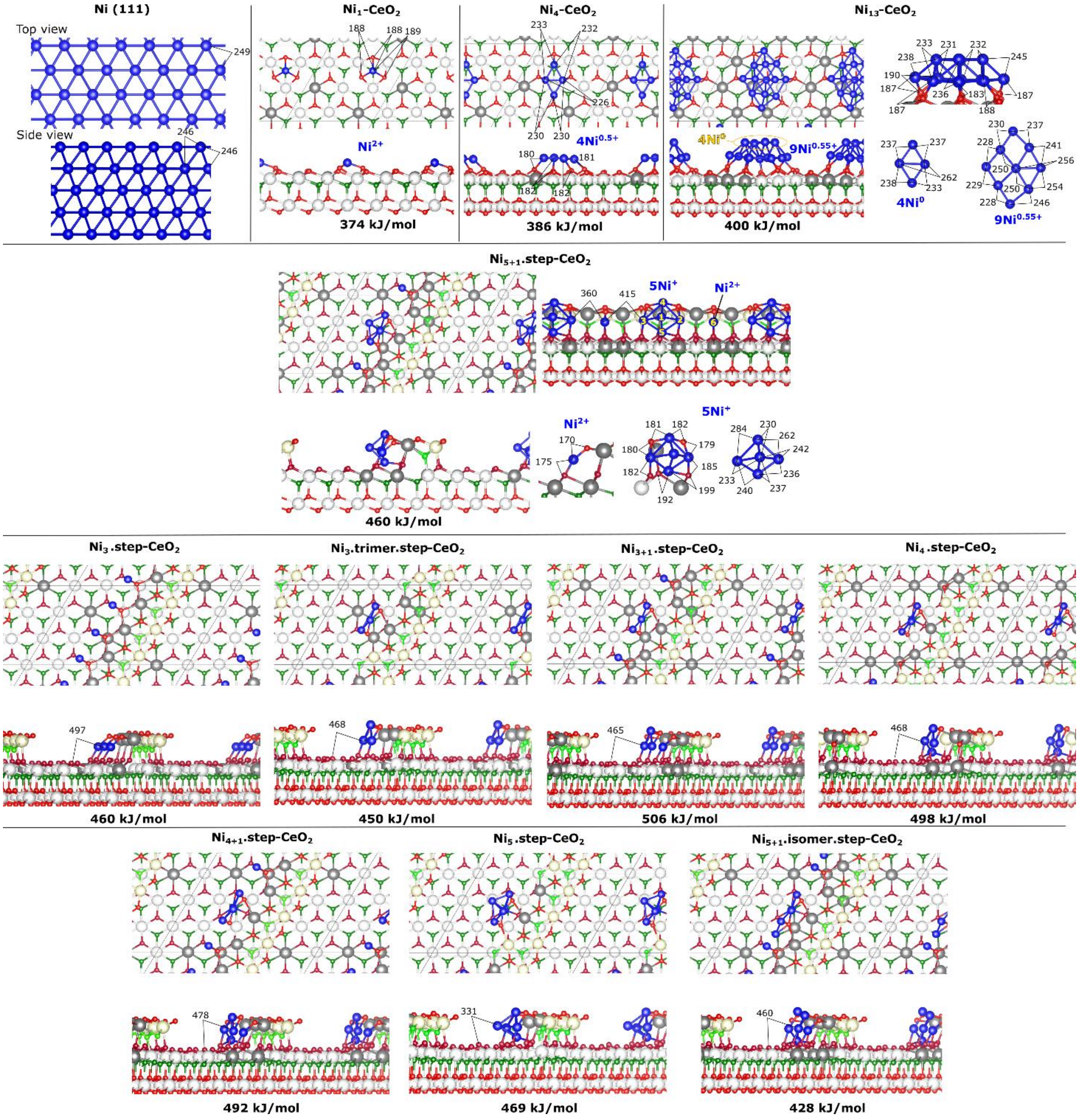

Figure S1. Models of Ni$_n$-CeO$_2$(111) (n=1, 4 and 13) systems and Ni$_3$, Ni$_3$.trimer, Ni$_3$.trimer+Ni$_1$ (Ni$_{3+1}$.step), Ni$_4$, Ni$_4$+N$_1$ (Ni$_{4+1}$.step), Ni$_5$ and Ni$_5$+N$_1$ (Ni$_{5+1}$.step) at a < 110>-type step. Ni$_{5+1}$.isomer.step is a structural Ni$_{5+1}$.step isomer. Surface/subsurface oxygen atoms in the outermost O–Ce–O trilayer are depicted in red/green, Ce^{4+} in white, and Ce^{3+} in gray. Values of the integral heat of adsorption of Ni$_n$ species are listed below each structure, in kJ/mol per Ni atom (relative to Ni gas). Selected bond lengths in pm are indicated. The Ni(111) surface is included for comparison. The atom numbering in the Ni$_{5+1}$.step-CeO$_2$ structure corresponds to that in Table S6 (#1 at the apex, #2–5 in the base, #6 isolated).

The energies in Figure S1 show that the $Ni_{5.step}$ cluster alone is 23 kJ/mol of Ni (or 115 kJ/mol total) less stable than the $Ni_{4+1.step}$ structure, so that the $Ni_{5.step}$ cluster alone would spontaneously decompose into the $Ni_{4+1.step}$ structure. This explains why we needed to study the $Ni_{5+1.step}$ structure in order to study a Ni_5 cluster at a step. The added Ni monomer at the step (which is separate from the Ni_5 cluster) simply maintains the stability of the cluster against dissociation, but did not have any apparent effects on the reaction of the Ni_5 step-edge cluster with methane (see below).

System	E_{IS}	E_{FS}	E_{FS}-E_{FS}[Metal]	E_{TS}		$E_{Barrier}$		ΔE_{TS}=
				Predicted	Calculated	Predicted	Calculated	$\Delta E_{Barrier}$
Ni(111)	-25.0	-33.6	0.0	77.8	61.4	102.7	86.4	-16.3
Ni_1-CeO_2(111)	-39.4	-41.3	-7.7	72.0	44.2	111.4	83.6	-27.8
Ni_4.2D-CeO_2(111)	-23.0	-99.8	-66.2	32.6	-9.6	55.7	13.4	-42.2
Ni_{13}.i-CeO_2(111)	-42.2	-11.5	22.1	92.2	-9.6	134.4	32.6	-101.8
Ni_{13}.t-CeO_2(111)	-34.6	-32.6	1.0	77.8	0.0	112.3	34.6	-77.8
Ni_3.step-CeO_2	-46.1	-47.4	-13.8	70.1	67.6	116.2	113.7	-2.5
Ni_{5+1}.step-CeO_2	-78.7	-129.6	-96.0	-13.4	-70.1	65.3	8.6	-56.7

Table S2. Calculated (no ZPE correction) energies (in kJ/mol) for the initial, E_{IS}, final, E_{FS}, and transition states, E_{TS}, for the CH_4(gas) → CH_3 + H reaction on Ni_n (n = 1, 4, 13) on CeO_2(111) terraces and Ni_3 and Ni_5+N_1 (Ni_{5+1}) at a < 110>-type step, as well as the (111) surface. Ni_{13}.t and Ni_{13}.i denote dissociation on a terrace and at an interface site of the Ni_{13}-CeO_2 system, respectively. All energies are relative to CH_4 in the gas phase and the corresponding clean systems. The predicted E_{TS} values correspond to the values obtained using the linear scaling relation E_{TS}= (0.67 E_{FS} +1.04) for the actual calculated final state, $E_{FS.}$ The predicted $E_{Barrier}$ values correspond to the activation energy barrier calculated as the energy difference between the predicted energy of the transition state and the calculated energy of the initial state. The model catalysts whose E_{TS} energy is less than zero is related to the fact that on them, CH_4 binds relatively strongly, so that if the barrier for the first H abstraction from the chemisorbed CH_4 molecule is sufficiently low, E_{TS} will be negative when referenced to gas-phase CH_4 and the clean surface.

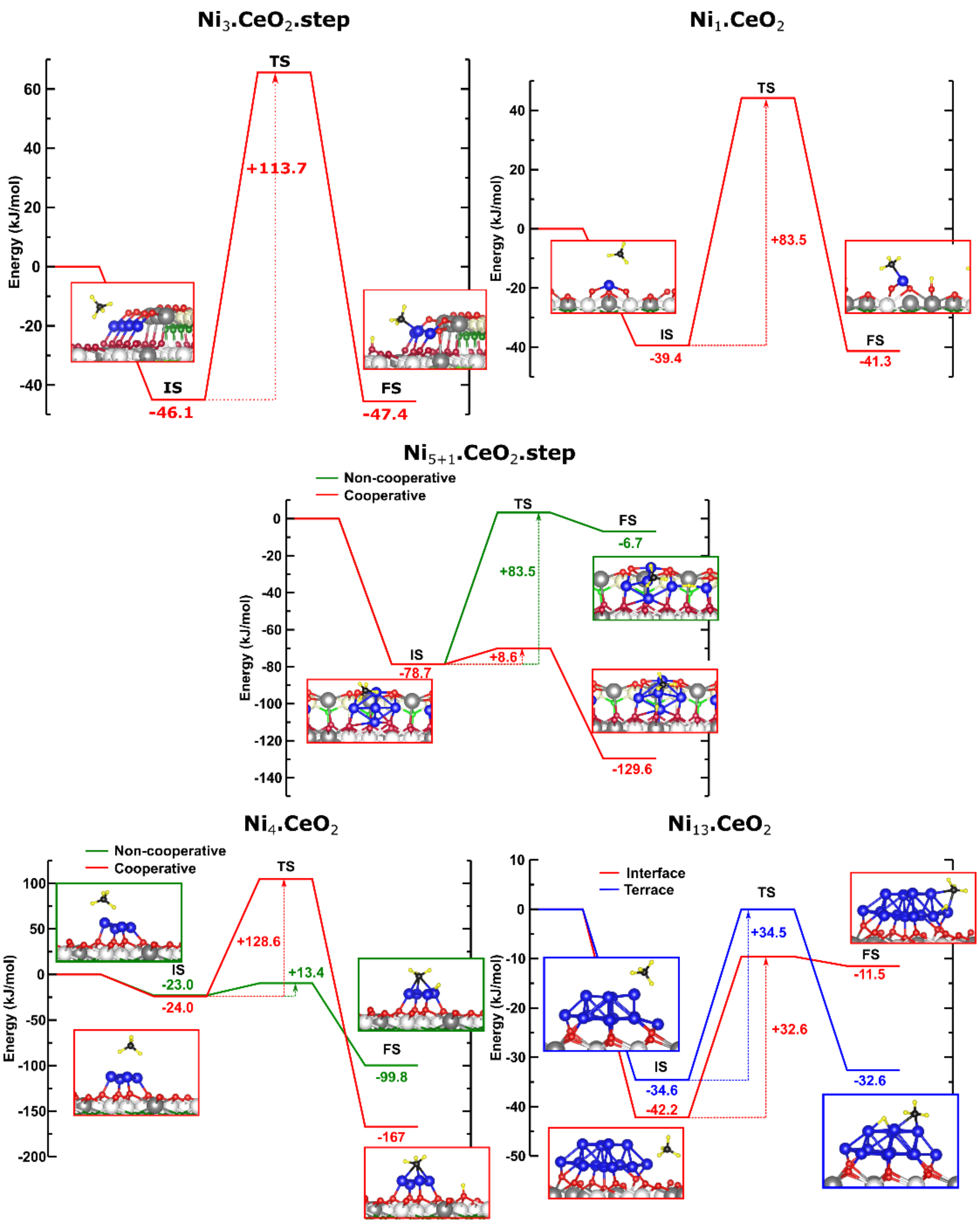

Figure S2. Lowest energy paths for the $CH_4 \rightarrow CH_3$+H reaction over the Ni/CeO_2 systems studied. For the Ni_5 pyramid at a <110> ceria step, the lowest energy path involves cooperative interactions between a metal cation and an O center, where hydrogen binds to a surface oxygen and methyl is adsorbed at the apex of the Ni_5 pyramid (no ZPE correction). The dissociation products correspond to stable chemisorbed states geometrically close to the corresponding transition state (TS), which we show in Figure S3. For the Ni_{13}-CeO_2 system the lowest energy states of CH_3 and H bound to Ni_{13} are shown in Figure S6.

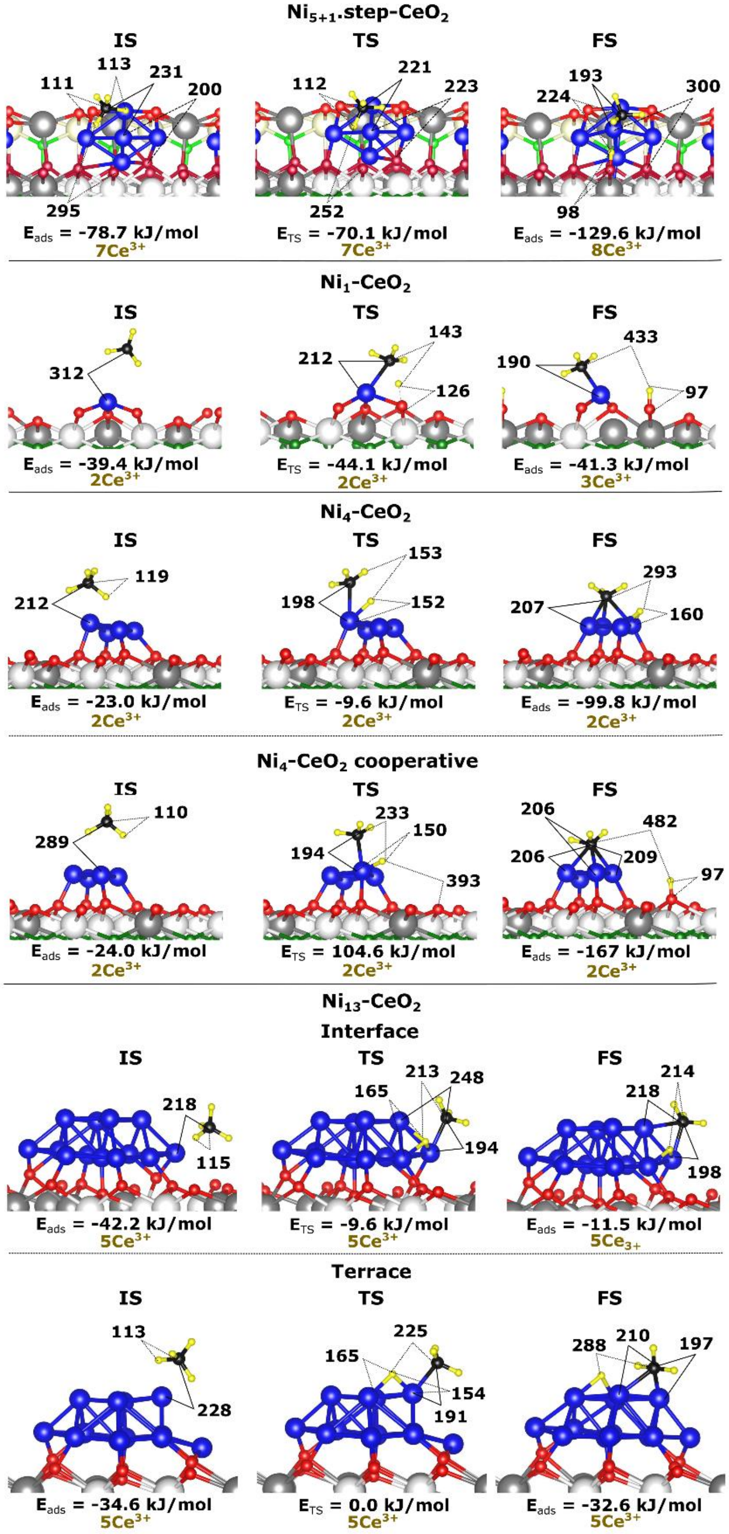

Figure S3. Initial state (IS), transition state (TS), and final state (FS) for the $CH_4 \rightarrow CH_3$+H reaction on the ceria-supported Ni_{5+1} aggregate at a < 110>-type step and the Ni_1, Ni_4 .2D and Ni_{13} clusters on CeO_2(111) terraces. Selected interatomic distances (in pm) are indicated.

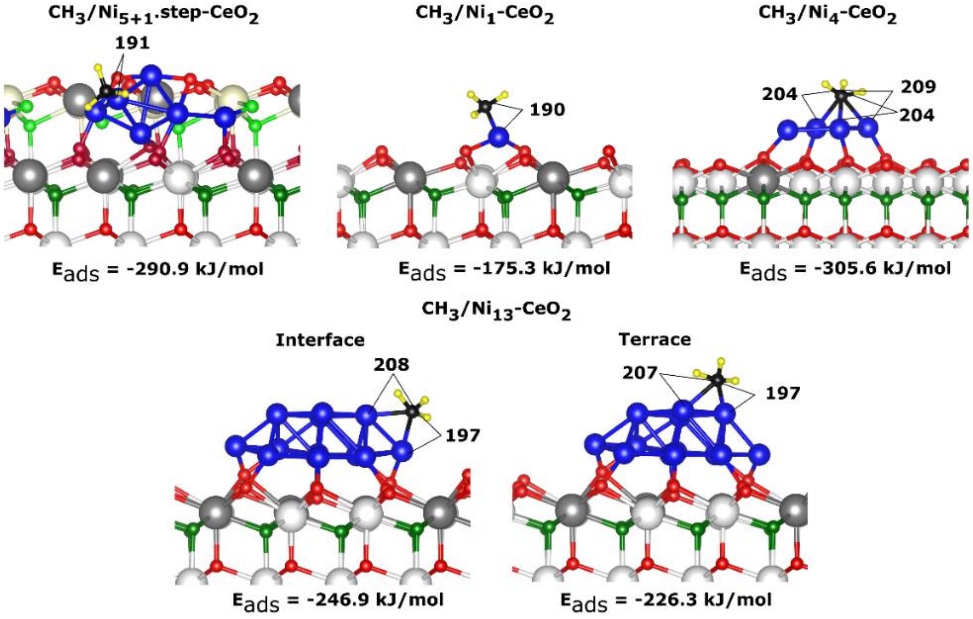

Figure S4. Binding of CH_3 on the ceria-supported Ni_{5+1} aggregate at a < 110>-type step and the Ni_1, Ni_4 .2D and Ni_{13} clusters on $CeO_2(111)$ terraces. Selected interatomic distances (in pm) are indicated. Adsorption energies are relative to CH_3 in the gas phase and the corresponding methyl-free systems.

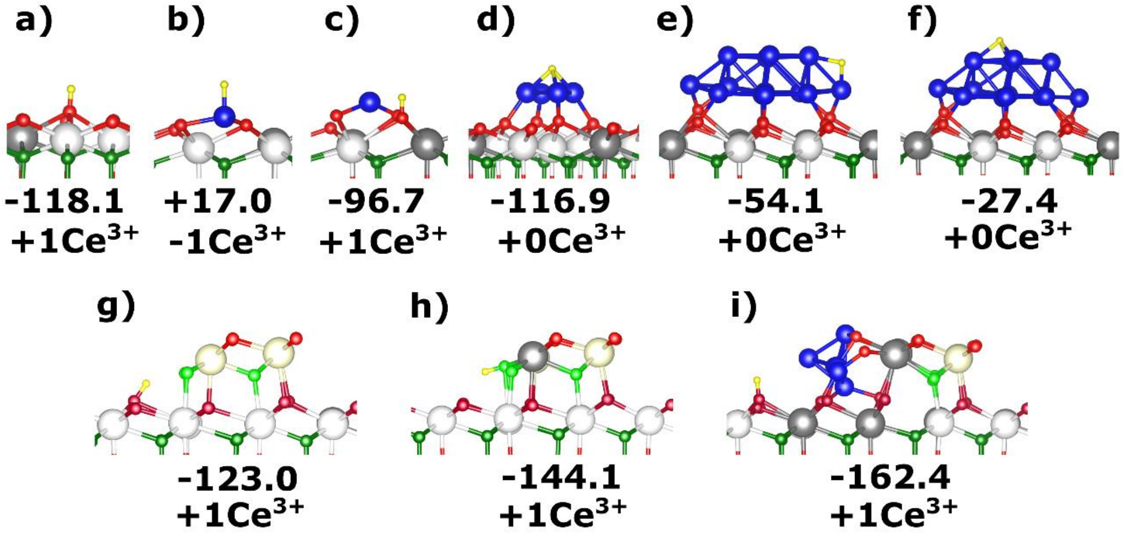

Figure S5 Adsorption of atomic H on various systems (kJ/mol wrt ½ H_2). The number of Ce^{3+} correspond to the changes upon hydrogen adsorption (ΔCe^{3+}). For the case of adsorption on a Ni^{2+} adatom (b) with initially $2\times Ce^{3+}$, the H/Ni_1-CeO_2 system has 1 Ce^{3+} and thus $\Delta Ce^{3+} = -1$.

System	H Binding Energy (kJ/mol wrt ½ H_2)	
	O–H	Ni–H
Ni(111)	-	-60.7
(a) CeO_2(111)	-118.1	-
(b-c) Ni_1-CeO_2	-96.70	+17.0
(d) Ni_4-CeO_2	-	-116.9
(e) Ni_{13}.i-CeO_2	-	-54.1
(f) Ni_{13}.t-CeO_2	-	-27.4
(g) O.t.step-CeO_2	-123.0	-
(h) O.s.step-CeO_2	-144.1	-
(i) Ni_{5+1}.step-CeO_2	-162.4	-

Table S3. Hydrogen binding energy on selected systems (see Figure S5). Ni_{13}.t and Ni_{13}.i denote adsorption on a terrace and at an interface site of the Ni_{13}-CeO_2 system, respectively, and O.t. step-CeO_2 and O.s. step-CeO_2 denote adsorption on an O atom at a terrace and at the step edge of the <110> step-CeO_2 system, respectively.

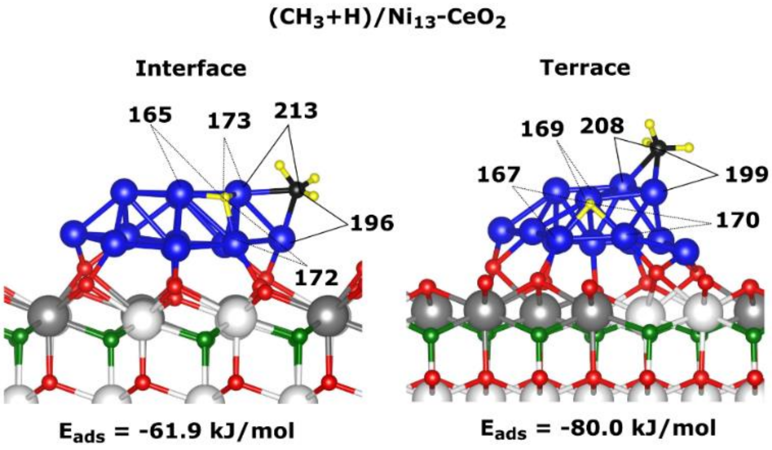

Figure S6. Lowest energy states for the adsorbed CH_3+H products of the first hydrogen abstraction from CH_4 on the Ni_{13} clusters supported on CeO_2(111) terraces. Selected interatomic distances (in pm) are indicated.

System M		CH_4.gas	CH_4/M-CeO_2	CH_4/M-CeO_2–CH_4.gas
Ni_{5+1}.step	C	4.0076	4.1291	0.12
	H	1.0033	0.9340	-0.07
	H	0.9871	1.0147	0.03
	H	0.9845	0.9371	-0.05
	H	0.9884	0.9452	-0.04
	Total	7.9709	7.9601	-0.01
Ni_1	C	4.0076	3.9129	-0.09
	H	1.0033	0.9943	-0.01
	H	0.9871	1.0393	0.05
	H	0.9845	1.0533	0.07
	H	0.9884	1.0049	0.02
	Total	7.9709	8.0047	0.03
Ni_4.2D	C	4.0076	4.1650	0.16
	H	1.0033	0.9304	-0.07
	H	0.9871	0.9965	0.01
	H	0.9845	0.9465	-0.04
	H	0.9884	0.9364	-0.05
	Total	7.9709	7.9748	0.00
Ni_{13}.i	C	4.0076	4.1440	0.14
	H	1.0033	0.9161	-0.09
	H	0.9871	0.9384	-0.05
	H	0.9845	0.9625	-0.02
	H	0.9884	1.0179	0.03
	Total	7.9709	7.9789	0.01
Ni_{13}.t	C	4.0076	4.1166	0.11
	H	1.0033	0.9432	-0.06
	H	0.9871	0.9975	0.01
	H	0.9845	0.9722	-0.01
	H	0.9884	0.9446	-0.04
	Total	7.9709	7.9741	0.00

Table S4. Bader charges (Q, in |*e*|) for a CH_4 molecule in the gas phase (CH_4.gas) and molecularly adsorbed (CH_4/system-CeO_2) on the ceria-supported Ni_{5+1} aggregate at a < 110>-type step and the Ni_1, Ni_4 .2D and Ni_{13} clusters on CeO_2(111) terraces. Ni_{13}.t and Ni_{13}.i denote adsorption on a terrace and at an interface site of the Ni_{13}-CeO_2 system, respectively. Bader charge differences (CH_4/system-CeO_2–CH_4.gas) are also listed.

System	Band	Empty States			Difference of Empty States	
		M.gas	M.CeO_2	CH_4/M.CeO_2	M.CeO_2–M.gas	CH_4/M.CeO_2–M.CeO_2
Ni_{5+1}.step	dz^2	0.13	0.16	0.07	0.03	-0.09
	dxy	0.15	0.31	0.16	0.16	-0.15
	dxz	0.25	0.18	0.23	-0.07	0.05
	dyz	0.15	0.15	0.18	0.00	0.03
	dx^2-y^2	0.22	0.07	0.07	-0.15	0.00
	dtotal	0.90	0.87	0.71	-0.03	-0.16
Ni_1	dz^2	0.48	0.00	0.00	-0.48	0.00
	dxy	0.03	0.26	0.31	0.23	0.05
	dxz	0.09	0.28	0.24	0.19	-0.04
	dyz	0.11	0.28	0.32	0.17	0.04
	dx^2-y^2	0.44	0.26	0.25	-0.18	-0.01
	dtotal	1.16	1.08	1.11	-0.08	0.03

Table S5. Integration of the projected density of states onto the d-states of the Ni atom at apex of the Ni_5 pyramid of the Ni_{5+1}.step system within the 0 (E_F) to +1.0 eV (+0.50 eV for Ni_1) energy interval. M.gas corresponds to the free-standing Ni_{5+1} aggregate resulting from the removal of the CeO_2 support from Ni_{5+1}.step-CeO_2, without further geometry optimization. M.CeO_2 corresponds to the results for the Ni_{5+1}.step-CeO_2 system, and CH_4/M-CeO_2 to those for the CH_4 adsorption on Ni_{5+1}.step-CeO_2.

System M	Atom	M-CeO_2	M.gas	M-CeO_2–M.gas	CH_4/M-CeO_2	CH_4/M-CeO_2– M-CeO_2
**Ni_{5+1}.step**	**Ni1**	**15.8217**	**15.9263**	**-0.10**	**15.6392**	**-0.18**
	Ni2	15.4463	16.0075	-0.56	15.5135	0.07
	Ni3	15.4218	16.0122	-0.59	15.4410	0.02
	Ni4	15.3345	15.9672	-0.63	15.4091	0.07
	Ni5	15.4586	15.9683	-0.51	15.5161	0.06
	Ni6	15.2655	16.1185	-0.85	15.3067	0.04
	Total	92.7484	96.0000	-3.25	92.8256	0.08
**Ni_1**	**Ni1**	**15.0559**	**16.0000**	**-0.94**	**15.0302**	**-0.03**
**Ni_4.2D**	Ni1	15.6472	16.1172	-0.47	15.7271	0.08
	Ni2	15.7281	16.1123	-0.38	15.7405	0.01
	**Ni3**	**15.7752**	**15.8868**	**-0.11**	**15.6519**	**-0.12**
	Ni4	15.7896	15.8838	-0.09	15.7701	-0.02
	Total	62.9401	64.0001	-1.06	62.8896	-0.05
**Ni_{13}.i**	Ni1	15.7760	15.9926	-0.22	15.7653	-0.01
	Ni2	15.7686	15.9945	-0.23	15.7778	0.01
	Ni3	15.7584	16.0759	-0.32	15.7583	0.00
	Ni4	15.6206	15.8241	-0.20	15.6629	0.04
	Ni5	15.7271	15.9925	-0.27	15.7089	-0.02
	Ni6	15.7570	15.9845	-0.23	15.7143	-0.04
	Ni7	15.7783	16.0961	-0.32	15.7462	-0.03
	**Ni8**	**15.4933**	**16.0833**	**-0.59**	**15.6272**	**0.13**
	Ni9	15.7255	16.0713	-0.35	15.7182	-0.01
	Ni10	16.0614	15.9764	0.08	16.0527	-0.01
	Ni11	16.0312	15.9647	0.07	16.0160	-0.02
	Ni12	16.0361	15.9785	0.06	16.0445	0.01
	Ni13	15.9869	15.9656	0.02	15.9949	0.01
	Total	205.5204	208.0000	-2.48	205.5872	0.07
**Ni_{13}.t**	Ni1	15.7760	15.9926	-0.22	15.8074	0.03
	Ni2	15.7686	15.9945	-0.23	15.7851	0.02
	Ni3	15.7584	16.0759	-0.32	15.7692	0.01
	Ni4	15.6206	15.8241	-0.20	15.6622	0.04
	Ni5	15.7271	15.9925	-0.27	15.7292	0.00
	Ni6	15.7570	15.9845	-0.23	15.7608	0.00
	Ni7	15.7783	16.0961	-0.32	15.7943	0.02
	Ni8	15.4933	16.0833	-0.59	15.4587	-0.03
	Ni9	15.7255	16.0713	-0.35	15.7384	0.01
	Ni10	16.0614	15.9764	0.08	16.0543	-0.01
	Ni11	16.0312	15.9647	0.07	16.0269	0.00
	Ni12	16.0361	15.9785	0.06	16.0468	0.01
	**Ni13**	**15.9869**	**15.9656**	**0.02**	**15.8968**	**-0.09**
	Total	205.5204	208.0000	-2.48	205.5301	0.01

Table S6. Bader charges (Q, in |*e*|) of the Ni atoms of a ceria-supported Ni_{5+1} aggregate at a < 110>-type step and of Ni_1, Ni_4 .2D and Ni_{13} clusters on CeO_2(111) terraces (M-CeO_2), see Figure S1. For Ni, 16 electrons ($3p^6$, $3d^8$, $4s^2$) were considered as valence. The charges upon adsorption of CH_4 is also listed. The atom on which CH_4 adsorbs is indicated in bold. Ni_{13}.t and Ni_{13}.i denotes adsorption on a terrace and at an interface site of the Ni_{13}-CeO_2 system, respectively. M.gas corresponds to the free-standing systems resulting from the removal of the CeO_2 support from M-CeO_2, without further geometry optimization. Bader charge differences (M-CeO_2–M.gas and CH_4/M-CeO_2– M-CeO_2) are also listed.

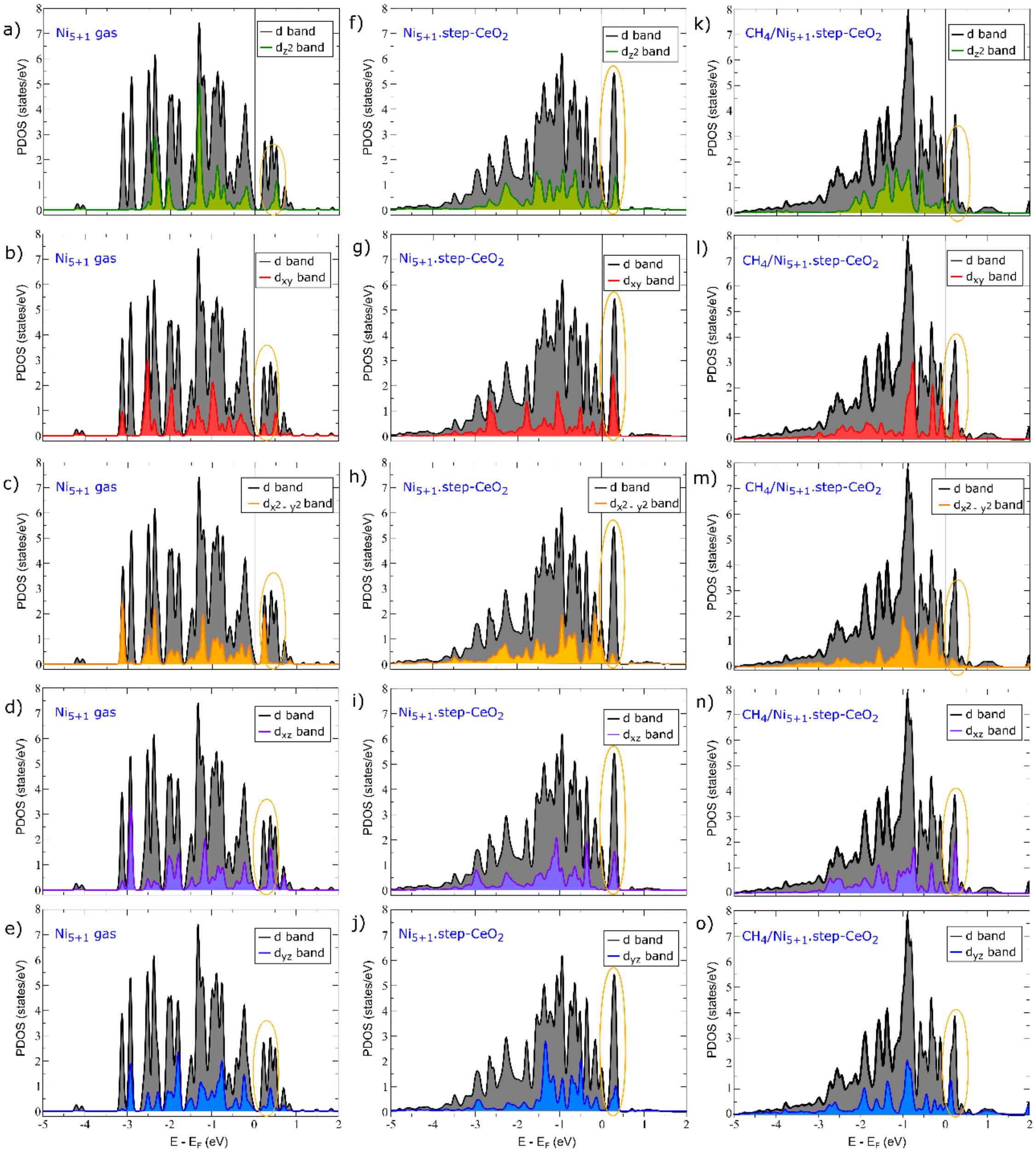

Figure S7. Projected density of states (PDOS) onto the d-states of the Ni atom at the apex of the Ni_5 pyramid of the Ni_{5+1}.step system. The energy zero is the Fermi level (E_F). The green, red, orange, violet and blue filled curves are the corresponding dz^2, dxy, dx^2-y^2, dxz, and dyz projected density of states, respectively. a-e) show the results for a free-standing Ni_{5+1} aggregate resulting from the removal of the CeO_2 support from Ni_{5+1}.step-CeO_2, without further geometry optimization, f-j) corresponds to the results for the Ni_{5+1}.step-CeO_2 system, and k-o) shows those for the CH_4 adsorption on Ni_{5+1}.step-CeO_2. The empty states close to E_F are highlighted.

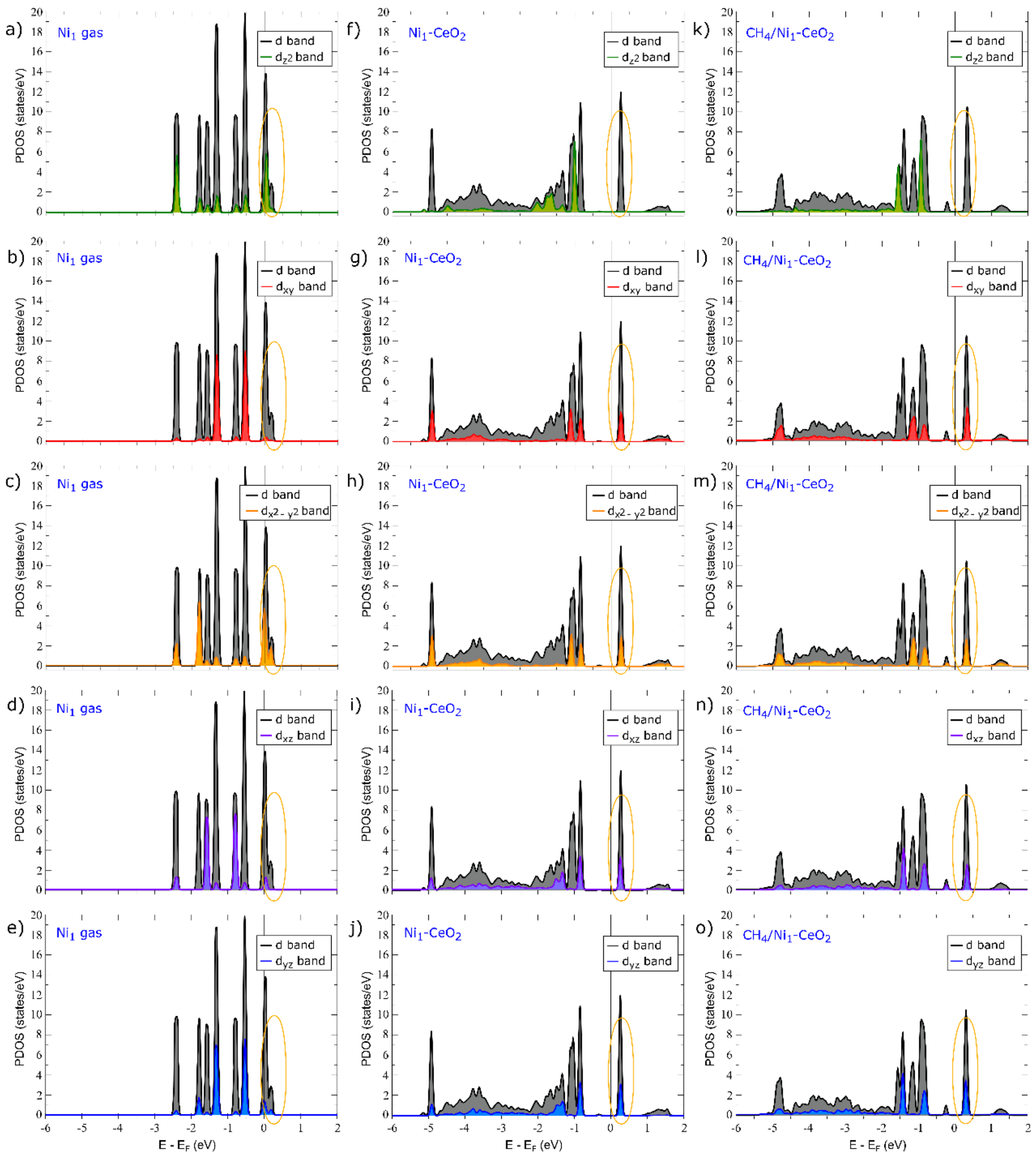

Figure S8. Projected density of states (PDOS) onto the d-states of a Ni atom on a $CeO_2(111)$ terrace. The energy zero is the Fermi level (E_F). The green, red, orange, violet and blue filled curves are the corresponding dz^2, dxy, dx^2-y^2, dxz, and dyz projected density of states, respectively. a-e) show the results for a free-standing Ni_1 aggregate resulting from the removal of the CeO_2 support from Ni_1 -CeO_2, without further geometry optimization, f-j) corresponds to the results for the Ni_1-CeO_2 system, and k-o) shows those for the CH_4 adsorption on Ni_1-CeO_2. The empty states close to E_F are highlighted.

We further note that for many of the systems in the original set in ref. [7], CH_4 is barely or not adsorbed and thus $E_{Barrier}$ (E_{TS}-E_{IS})= E_{TS} and $E_{Reaction}$ (E_{FS}-E_{IS}) = E_{FS}. However, this is not true for some of the metal-CeO_2 systems (nor for IrO_2(110) and Pd(101)), for which the binding of the initial state is substantial with one C-H bond partially activated, as discussed above. Figure S9 displays the activation energy as a function of the reaction energy for selected systems. The comparison between the best linear fit for the $E_{Barrier}$ vs. $E_{Reaction}$ data corresponding to the Ni_4.2D, Pt_4.2D, Co_4.2D, and Ni_{13} clusters on terraces and Ni_{5+1} at steps and that for the E_{TS} vs. E_{FS} in Figure 3 of the main text, indicates that 60% of the slope of the E_{TS} vs. E_{FS} regression line is due to a "true" Brønsted relation, and 40% is due to the fact that the final state energy tracks to some extent the initial state energy. This 40% is due to the simple fact that metal sites that strongly bind one small C/H containing adsorbate also tend to bind other C/H containing adsorbates strongly.

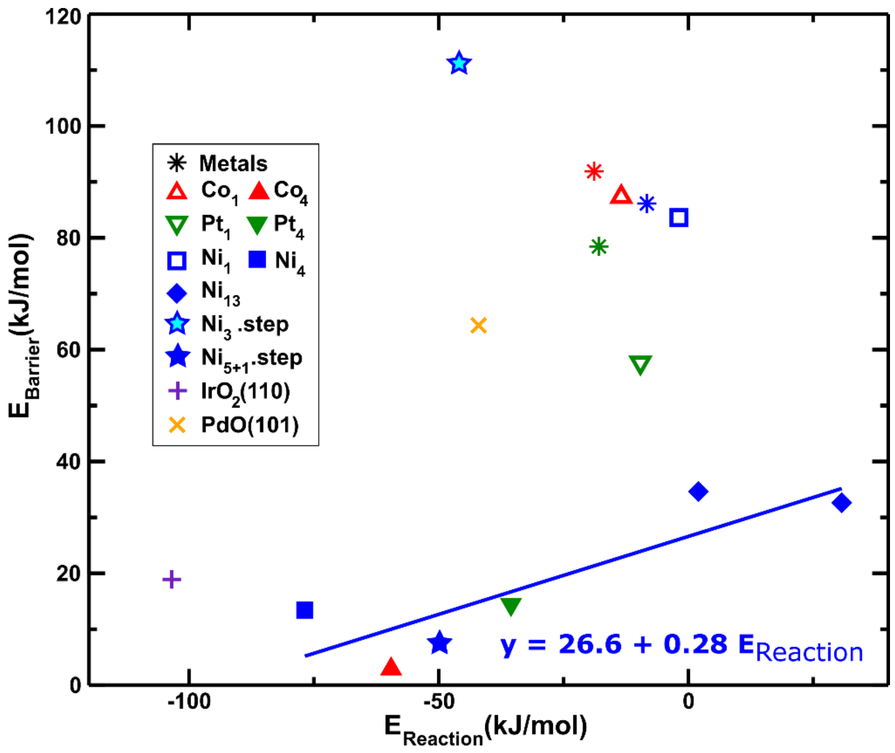

Figure S9. Brønsted relation between the calculated activation barrier ($E_{Barrier}$) and reaction energy ($E_{Reaction}$) for the surface-stabilized pathway in methane dissociation on a variety of materials. The purple (+) and yellow (x) symbols are for IrO_2(110) and Pd(101) presented in ref. [8,9] and ref. [10], respectively. The green, red and blue symbols correspond to the values for M_1 atoms and M_4 clusters (M= Pt, Co, Ni) on the CeO_2(111), respectively. Values for the extended Pt(111), Co(0001) and Ni(111) surfaces, as in ref. [2], as well as on the Ni_{13} clusters on terraces and Ni_3 and Ni_{5+1} at steps are shown. The blue line is the best linear fit for the data corresponding to the Ni_4.2D, Pt_4.2D, Co_4.2D, and Ni_{13} clusters on terraces and Ni_{5+1} at steps.

References

1 Liu, Z. *et al.* In Situ Investigation of Methane dry reforming on Metal/Ceria(111) surfaces: Metal–Support Interactions and C−H Bond Activation at Low Temperature. *Angew. Chem. Int. Ed.* **56**, 13041-13046, doi:10.1002/anie.201707538 (2017).

2 Lustemberg, P. G. *et al.* Breaking simple scaling relations through metal–oxide interactions: Understanding room-temperature activation of methane on M/CeO_2 (M = Pt, Ni, or Co) interfaces. *J. Phys. Chem. Lett.* **11**, 9131-9137, doi:10.1021/acs.jpclett.0c02109 (2020).

3 Monkhorst, H. J. & Pack, J. D. Special points for Brillouin-zone integrations. *Phys. Rev. B* **13**, 5188-5192, doi:10.1103/PhysRevB.13.5188 (1976).

4 Janthon, P. *et al.* Bulk properties of transition metals: A challenge for the design of universal density functionals. *J. Chem. Theory Comput.* **10**, 3832-3839, doi:10.1021/ct500532v (2014).

5 Zhang, G.-X., Reilly, A. M., Tkatchenko, A. & Scheffler, M. Performance of various density-functional approximations for cohesive properties of 64 bulk solids. *New J. Phys.* **20**, 063020 (2018).

6 Henkelman, G., Uberuaga, B. P. & Jonsson, H. A climbing image nudged elastic band method for finding saddle points and minimum energy paths. *J. Chem. Phys.* **113**, 9901-9904, doi:10.1063/1.1329672 (2000).

7 Latimer, A. A. *et al.* Mechanistic insights into heterogeneous methane activation. *Phys. Chem. Chem. Phys.* **19**, 3575-3581, doi:10.1039/c6cp08003k (2017).

8 Kim, M. *et al.* Adsorption and oxidation of CH_4 on oxygen-rich IrO_2(110). *J. Phys. Chem. C* **123**, 27603-27614, doi:10.1021/acs.jpcc.9b08215 (2019).

9 Liang, Z., Li, T., Kim, M., Asthagiri, A. & Weaver, J. F. Low-temperature activation of methane on the IrO_2(110) surface. *Science* **356**, 299-303, doi:10.1126/science.aam9147 (2017).

10 Antony, A., Asthagiri, A. & Weaver, J. F. Pathways and kinetics of methane and ethane C-H bond cleavage on PdO(101). *J Chem Phys* **139**, 104702, doi:10.1063/1.4819909 (2013).
